# A PCR Method That Can Be Further Developed into PCR-RFLP Assay for Eight Animal Species Identification

**DOI:** 10.1155/2018/5890140

**Published:** 2018-02-05

**Authors:** Feng Guan, Yu-Ting Jin, Jin Zhao, Ai-Chun Xu, Yuan-Yuan Luo

**Affiliations:** College of Life Sciences, China Jiliang University, Hangzhou 310018, China

## Abstract

There are many PCR-based methods for animal species identification; however, their detection numbers are limited or could not identify unknown species. We set out to solve this problem by developing a universal primer PCR assay for simultaneous identification of eight animal species, including goat, sheep, deer, buffalo, cattle, yak, pig, and camel. In this assay, the variable lengths of mitochondrial DNA were amplified using a pair of universal primers. PCR amplifications yielded 760 bp, 737 bp, 537 bp, 486 bp, 481 bp, 464 bp, 429 bp, and 359 bp length fragments for goat, sheep, deer, buffalo, cattle, yak, pig, and camel, respectively. This primer pair had no cross-reaction with other common domestic animals and fish. The limit of detection varied from 0.01 to 0.05 ng of genomic DNA for eight animal species in a 20 *µ*l PCR mixture. Each PCR product could be further digested into fragments with variable sizes and qualitative analysis by *Ssp*I restriction enzyme. This developed PCR-RFLP assay was sufficient to distinguish all targeted species. Compared with the previous published related methods, this approach is simple, with high throughput, fast processing rates, and more cost-effective for routine identification of meat in foodstuffs.

## 1. Introduction

The consumption of meat and meat products is increasing each year in the world. On the other hand, food authenticity issues in the form of adulteration and improper description have existed for as long as food has been offered for sale. In China, “hang a sheep head, sell vinegar” is a widely known proverb. However, meat adulteration affects food safety, quality, and many other respects, becoming a public focus recently. Since the 1980s, many immunological and molecular methods for species identification in food products have been developed [1–8]. Concomitant with advances in large-scale integrated molecular technology, DNA analysis methods have been in rapid development. Many assays based on DNA analysis, especially PCR-based methods, have been developed for species identification in foodstuffs. Though each of these methods has its own advantages and disadvantages, the number of species identified in a single PCR was limited to 4-5 [9]. Compared with other PCR methods, the combination of universal primer PCR and restriction fragment length polymorphism (RFLP) assays has advantages of increased simplicity, specificity, and sensitivity and has been used in many meat identification practices [10–17]. On the other hand, a few modified PCR assays combining universal primer with specific primer or RFLP had been developed [18, 19]; these methods increased efficiency and versatility. Furthermore, PCR-RFLP had additional advantages over multiplex PCR and species-specific PCR, such as the ability to detect a larger number of animal species and differentiate between closed animal species.

With the development of Western China, more and more local meat products of mutton, beef, yak, deer, and camel have been sold to all parts of the country. There have been a few analytical methods for the identification of yak [20, 21], deer [17], buffalo [22, 23], and camel [24] meat in food products, but to our knowledge, there is no one method for simultaneously identifying two and more animal species, for goat, sheep, buffalo, cattle, yak, deer, and camel. Furthermore, pork is popular with Chinese people, but Muslim populations are widely distributed in China; all the meat products from pork source are forbidden in halal foods. In this regard, precise identification of meat origin has become a vital element in food quality control procedures. Thus, the development of reliable, simple, and sensitive analytical methods for evaluating meat authenticity is of critical importance.

In recent years, universal primer PCRs have been used and further developed for animal species identification combination with other DNA analysis methods [19, 25–29], which had many advantages over common and even real-time PCR assays. In this study, a new pair of universal primers was designed and a species identification assay has been developed using the variable size fragments, and this assay can be further developed into PCR-RFLP assay, which showed potential as a tool for cost-effective, rapid, specific, and sensitive detection of goat, sheep, buffalo, cattle, yak, deer, pig, and camel simultaneously.

## 2. Materials and Methods

### 2.1. Samples Collection and DNA Extraction

The samples of known species origins including goat *(Capra aegagrus hircus)*, sheep *(Ovis aries)*, deer *(Cervus axis)*, buffalo *(Bovinae)*, cattle *(Bos primigenius)*, yak *(Bos mutus grunniens)*, pig *(Sus scrofa)*, camel *(Pilus cameli)*, horse *(Equus caballus)*, donkey *(Equus asinus)*, rabbit *(Oryctolagus cuniculus)*, chicken *(Gallus gallus)*, duck *(Anas platyrhynchos)*, rat *(Rattus norvegicus)*, dog *(Canis lupus familiaris)*, frog *(Rana catesbiana)*, and fish *(Carassius auratus)* were collected from a slaughterhouse and experimental animal center in Hangzhou, China, and were stored at −20°C in our laboratory. In addition, fifteen commercial meat product samples were purchased to assess this developed method in practice; the ingredients were labeled as deer, yak, camel, beef, and mutton, respectively.

Genomic DNA was extracted from meat samples using an Animal Tissue DNA Extraction Kit (Takara) according to supplied instructions. Following DNA extraction, the purity and concentration of all the DNA samples were confirmed using a UV-Vis spectrophotometer (NanoDrop 2000, Thermo). DNA samples were diluted to a final concentration of 10 ng/*µ*l and stored at 4°C for next use.

### 2.2. Universal Primers and PCR Amplification

The mitochondrial DNA (mtDNA) sequences of all the above animals were retrieved from the NCBI database and were aligned using ClustalW sequence alignment tool to select the congenerous conserved regions. Two regions were selected as primer design areas, and a pair of universal primers was designed to amplify variable length mtDNA sequences from genomic DNA of yak, deer, goat, sheep, pig, camel, cattle, and buffalo but no other animals. There were several insertion-deletion polymorphisms in the amplified sequences, which, in theory, could yield different length fragments for each animal. The primer sequences are as follows: forward primer (5′-CCTCCCTAAGACTCAGGGAA-3′) and reverse primer (5′-AGCGGGTTGCTGGTTTCACG-3′). The designed primers were also screened for unique specificity to check cross-species binding with other animal or plant species using the online BLAST local alignment tool in the NCBI database.

PCR amplifications were carried out using a MJ-200 thermal cycler in a total of 20 *µ*l mixture containing 2.0 *μ*l 10x PCR buffer, 1.2 *μ*l of dNTP mixture (25 mM), 1.6 *μ*l MgCL_2_ (25 mM), 1 U of *Taq* polymerase, 2 *μ*l each of universal primers (10 mM), and 3 *μ*l DNA template (about 30 ng). The PCR conditions consisted of preheating at 94°C for 5 min, followed by 30 cycles of denaturation at 94°C for 30 s, annealing at 61°C for 30 s, and extension at 72°C for 30 s. Then final extension was done at 72°C for 5 min. The 5.0 *µ*l PCR product obtained was analyzed on 2% agarose gel in 1x TAE buffer stained with 4S Red as a visualizing agent and run for 40 min at 90 V. A known DNA ladder Marker C (Shanghai Sangon Biotech) was electrophoresed simultaneously in order to assess the size of the amplification product.

### 2.3. Restriction Fragment Length Polymorphism (RFLP) Analysis

To further discriminate between targeted animals, expected nucleotide sequences were restriction mapped with the Mapdraw program of DNASTAR (NY, USA). After testing, one restriction enzyme *Ssp*I (TakaRa) was selected to digest the PCR products in separate reactions. Briefly, 5 *μ*l of the PCR product and 2 *μ*l of the restriction enzyme were prepared by mixing 2 *μ*l of 10x buffer, and sterile free water was added for a total volume of 20 *μ*l. The tubes were incubated at 37°C for approximately 4 h. The digested PCR products were then separated on 2.5% agarose gel.

### 2.4. PCR Products Sequencing and Commercial Samples Detection

To further validate this assay, each of the PCR products was purified and then sequenced in Hangzhou Qingke Biotechnology Company and then were analyzed using the BLAST local alignment tool. Fifteen commercial samples were screened using this developed assay, and each of the PCR products obtained was extracted and purified separately and then digested by the restriction enzyme *Ssp*I.

## 3. Results and Discussions

### 3.1. DNA Extraction and PCR Amplifications

**Figure 1:**
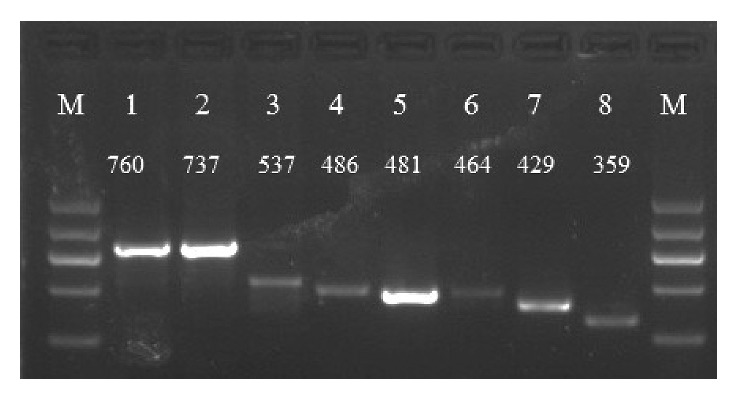
PCR amplification results for eight animal species. Lanes 1–8 represent goat, sheep, deer, buffalo, cattle, yak, pig, and camel, respectively. Lane M represents DNA marker; the lengths of different PCR products were noted above the band.

In the present study, all the genomic DNA samples were measured. The spectrophotometric assessment results showed that concentrations varied between 20 and 200 ng/*μ*l, and the purity (A_260_/A_280_ = 1.72–1.96) was suitable for PCR amplification. The universal primer pair generated 760 bp, 737 bp, 537 bp, 486 bp, 481 bp, 464 bp, 429 bp, and 359 bp length fragments from goat, sheep, deer, buffalo, cattle, yak, pig, and camel DNA templates, respectively (Figure 1).

### 3.2. PCR Specificity and Sensitivity

**Figure 2:**
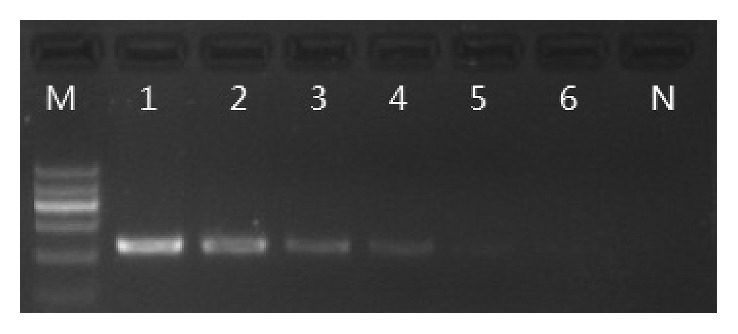
Sensitivity test for pig DNA. Lanes 1–6 represent the PCR products with serial dilution of pig DNA template. The content of pig DNA was 30.0, 10.0, 1.0, 0.1, 0.01, and 0.001 ng. Lane N was negative control.

The specificity of primers was checked against the extracted DNA samples from common animals, which included dog, chicken, horse, rat, donkey, rabbit, duck, frog, and fish. The specificity test results showed that no cross amplification was detected. The sensitivity was tested using serially diluted DNA samples from goat, sheep, deer, buffalo, cattle, yak, pig, and camel, and until the PCR products could not be visualized on 2.0% agarose gel. DNA band patterns (Figure 2) indicated that the detection limit of DNA from pigs was even at 0.001 ng, and the minimum detection levels of goat, sheep, deer, buffalo, cattle, yak, and camel DNA templates were between 0.01 and 0.05 ng.

### 3.3. PCR-RFLP Analysis and Sequencing

**Figure 3:**
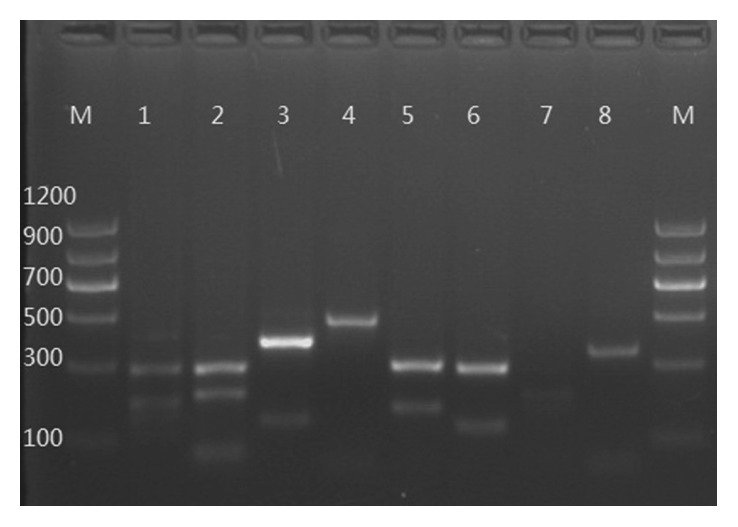
The PCR-RFLP result for eight target animals. Lane M represents DNA marker; lanes 1–8 represent goat, sheep, deer, buffalo, cattle, yak, pig, and camel, respectively.

Each of the PCR products yielded different patterns for eight animal species after *Ssp*I restriction enzyme digestion. The PCR products yielded 291 bp, 192 bp, 160 bp, and 118 bp for goat; 300 bp, 213 bp, and 75 bp for sheep; 391 bp and 146 bp for deer; 303 bp and 178 bp for cattle; 214 bp, 178 bp, and 73 bp for yak; and 300 bp and 129 bp for pig. However, the PCR products from buffalo and camel DNA could not be cleaved by *Ssp*I; thus, the PCR products were always 486 bp and 359 bp, respectively. The PCR product digestion patterns are shown in Figure 3. Unique restriction pattern of *Ssp*I for each species was found to satisfactorily differentiate between all eight animal species.

To confirm the developed method, each of the PCR products was purified and sequenced. The sequencing results indicated that the sizes and sequences of all the PCR products corresponded exactly to that of the expected amplicons. The similarity parameters were as high as 100% in accordance with the GenBank database. One exception was buffalo (98.2%). This difference was caused by two single nucleotide polymorphic loci detected in the referenced sequence of buffalo compared to GenBank Accession no. AY702618. The sequencing results were fully in accordance with the expected amplicons, which indicates that this developed assay had a higher specificity.

### 3.4. Commercial Samples Detection

**Figure 4:**
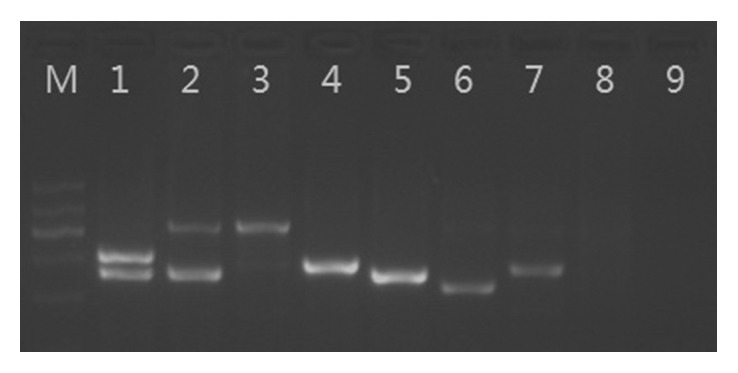
Identification results of commercial meat samples. Samples in lanes 1–7 were labeled as deer, mutton, mutton, beef, pork, camel, and yak meat. Lane 8 was soybean DNA, and lane 9 was negative control. But, the samples in lanes 1 and 2 contained pork.

The fifteen commercial meat products were detected using this developed assay, two animal ingredients were found in two of the fifteen samples, while other samples had only one ingredient (Figure 4). The two samples were labeled as deer meat and mutton, respectively. Judging from the electrophoretic profiles, these two meat products were adulterated with pork. To confirm this finding, all the electrophoretic bands were extracted and sequenced. Sequencing results confirmed that the two samples contained pork ingredients. The other thirteen samples contained only one meat ingredient and were consistent with the labeling meat species.

With the development of economy, meat products become a daily food for consumption. Furthermore, yak, camel, and deer meats are new popular meat products in the Chinese market, especially as leisure foods. The price of these meat products is higher than that of common meat products such as beef and pork. On the other hand, the rising price and decreasing availability of high-quality meat drive some meat producers to misrepresent and/or adulterate meat and meat products. In fact, food adulteration has been around for a long time. The most common economic fraudulence widely spread in meat industry is adulteration or substitution of costlier meat with cheaper or inferior meat. Preventing meat adulteration in foods is important for economic, religious, and health reasons. In addition, identification of the species origin in processed meat products is an important task in food hygiene, food codex, food control, and veterinary forensic medicine.

In order to protect consumers from fraud and adulteration, many meat and/or animal species identification methods have been developed to date. DNA assays have become common methods in practice because they have many advantages over other analyses. The stability of mtDNA is higher than that of genomic DNA and it is distributed in all the tissues. Therefore, mtDNA sequences are preferential for DNA barcoding in species identification [9, 30–37]. It is clear that PCR assay is a preferred technique based on DNA analysis for meat species identification to date. PCR and its derived techniques have been widely used for meat authentication, which included DNA hybridization, species-specific PCR, multiplex PCR, PCR-RFLP, PCR-SSCP, and PCR sequencing [2, 31, 38, 39]. Among these techniques, PCR-RFLP is considered as a highly discriminatory, reliable, and reproducibility method. Furthermore, the advantage of mitochondrial DNA-based PCR-RFLP analysis derives from the fact that there are many mitochondria per cell and many mitochondrial DNA molecules within each mitochondrion, making mtDNA a naturally amplified source of genetic variation [10, 14, 24, 32, 40, 41].

In the past few years, PCR-RFLP assays had been used to identify food animal species targeting CytB gene [14, 17, 42] and 12S rRNA gene [16, 43]. Among these assays, the same length fragments were amplified from two or more animals in the PCR procedure, then PCR products were digested using one or more restriction enzymes to yield different patterns [14, 15]. Partis et al. [34] developed a PCR-RFLP method for the detection of 22 animal species, but this method was unsuitable for analyzing meat mixtures. Several similar multiplex species identification assays had been reported. For example, Wang et al. [32] reported a terminal restriction fragment length polymorphism (T-RFLP) method for the identification of 12 animal species by targeting the 12S rRNA gene, but it required two restriction enzymes, double-fluorescently labeled primers and capillary electrophoresis. Hanapi et al. [44] reported a common primer multiplex PCR (CP-M-PCR) for the identification of pig, ruminant, avian, and rabbit meat, but this method could not distinguish closely related species, such as buffalo and cattle, and sheep and goat. There have been other several reported related methods based on PCR analysis, but these methods were often not suitable for mixture of food products, as the process was time consuming, inadequate, and expensive.

In the present study, a modified PCR assay was developed using universal primers for simultaneous identification of goat, sheep, deer, buffalo, cattle, yak, pig, and camel. Also, this assay could be further developed into RFLP method; the fragments of different lengths produced from PCR products could be identifying the animal species. This method can detect any targeting individual species within a meat mixture, which had better discrimination and specificity than many other multiple PCR methods.

This developed PCR-RFLP assay was a simple and rapid technology for identifying multiple species. This method did not require expensive equipment, and the optimization procedure was simple in comparison with multiplex PCR, real-time PCR, and other technologies that are used for the detection of two or more animal species. Interestingly, this method could identify individual species within a meat mixture by separating different size PCR products. This method was developed based on conventional PCR platform and required a common PCR thermal cycler, so it could be carried out in most laboratories. Furthermore, the developed PCR had no cross-reaction with DNA samples from common meats, such as duck, chicken, rat, frog, fish, donkey, and horse, and the PCR system had a high sensitivity at a minimum level of 0.001 ng of DNA. In general, the detection sensitivity for animal species such as poultry, ruminant, and pig using common PCR and real-time PCR was up to 0.1–1% of the ingredients and was also able to detect common meat species down to 0.15–0.01 ng of DNA [19, 45, 46]. So, it seems that the sensitivity is even higher than that of real-time PCR targeting for genomic DNA. This high sensitivity was probably because mtDNA was a multiple copy gene in each cell, and there was an increased probability of survival under different processing conditions. These advantages ensured that amplification was successful even in certain samples containing small amounts of DNA [47]. Although this developed PCR-RFLP had advantages over conventional PCR and multiplex PCR in some respects, it could not well satisfy the needs for meat products supervision in practice. Instead, this process could be combined with other species identification methods to get a better result in practice. For example, this method could combine with other species-specific primers to establish a multiplex PCR or combined with direct PCR to improve the speed [48, 49]. In fact, the choice of an appropriate method for species identification is decided by multiple considerations, such as sensitivity, specificity, accuracy, discriminatory ability, reproducibility, cost, speed, availability of equipment, and availability of suitably trained staff. The developed method provides more choices for animal identification, especially for meat food supervision.

## 4. Conclusion

In conclusion, a pair of universal primers for amplifying variable length fragments in eight animal species was designed and a PCR assay was developed, which could be further developed into PCR-RFLP. This developed method can be used to identify eight animal species. This method was specific, sensitive, and reliable in the simultaneous identification of goat, sheep, deer, buffalo, cattle, yak, pig, and camel. This proposed method was relatively simple and rapid, does not require expensive equipment, and could be performed in most laboratories. It is a practical approach for routine analysis to determine fraudulent and/or mislabeled substitution in meat products.
